# Alterations in mental health and quality of life among healthcare workers in times of COVID-19: Four-stage cross-sectional study during first four pandemic waves in Poland

**DOI:** 10.3389/fpsyt.2022.1027734

**Published:** 2022-11-24

**Authors:** Mateusz Babicki, Krzysztof Kowalski, Bogna Bogudzińska, Agnieszka Mastalerz-Migas

**Affiliations:** ^1^Department of Family Medicine, Wrocław Medical University, Wrocław, Poland; ^2^Division of Consultation Psychiatry and Neuroscience, Department of Psychiatry, Wrocław Medical University, Wrocław, Poland; ^3^Faculty of Medicine, Wrocław Medical University, Wrocław, Poland

**Keywords:** COVID-19, anxiety, depression, mental health, pandemic

## Abstract

**Background:**

The COVID-19 pandemic has had many unexpected effects that have affected the mental health of healthcare workers. In response to the crisis, healthcare workers appear to be the most vulnerable to the psychological effects of the pandemic. The purpose of the study was to assess the prevalence of depressive and anxiety symptoms and healthcare workers’ quality of life during the different stages of the COVID-19 pandemic.

**Materials and methods:**

The questionnaire was distributed in four stages corresponding to the different waves of the pandemic in Poland. The last stage of the study covered the period from November 1, 2021 to November 31, 2021 which coincided with the fourth wave of COVID-19 in Poland. The Beck Depression Inventory II (BDI-II), Generalized Anxiety Disorderd-7 (GAD-7), and Manchester Brief Assessment of Quality of Life (MANSA) scales were used.

**Results:**

A total of 1,243 respondents participated. A gradual increase in moderate and severe anxiety was observed as the pandemic continued, comparing waves I and IV of the pandemic. No statistically significant differences were observed in comparing the mean values of the BDI-II, GAD-7, and MANSA scales across waves. A decrease in fear due to the disease and neighbor’s quarantine was found. Women, single people and those with a psychiatric history are more likely to be affected by the destructive impact of the pandemic.

**Conclusion:**

The COVID-19 pandemic is significantly affecting the mental health and quality of life of healthcare workers, but trend is not uniform. It is necessary to continue monitoring the mental health of medical workers, who are the most important link in the fight against the pandemic.

## Introduction

The Coronavirus Disease-19 (COVID-19) pandemic has had many unexpected effects that have affected the mental health of the public, especially healthcare workers (1). In Poland, as in other European countries, its five waves have been distinguished until May 2022 (2). The variation in epidemiological characteristics in the different waves was due to the successive mutations of the virus that appeared. Wave four, which was dominated by the delta variant, was characterized by the greater transmission of the virus and more severe courses especially among the young and unvaccinated compared to the previous three (3).

In response to the ongoing crisis, healthcare workers appear to be the most vulnerable to the psychological effects of a pandemic. This has been confirmed in recent systematic reviews and meta-analyses, which confirm a significant escalation in the level of depression and anxiety associated with the pandemic among healthcare workers (4–8). The first such observations were conducted in China and showed that 50.7% of healthcare workers struggled with anxiety symptoms, while 44.7% struggled with depression (9). The possible reasons for the phenomenon were the rapid reorganization of the healthcare system, the increase in work intensity, and the increased likelihood of infecting oneself and loved ones (10, 11). In addition, the need to function under chronic stress has contributed to exacerbating the symptoms described above (12). There was also a positive correlation between anxiety and professional burnout and decreased quality of life (13). However, the impact was lessened over time, and some of the population adapted to the new reality. A retrospective study of nurses in China showed a declining trend in the incidence of the symptoms described above 1 month after the main peak of the disease (14). Similarly, observations from Belgium showed a reduction in depression and anxiety among frontline nurses, 2 months after the pandemic broke out (15). Convergent observations were made in Italy among healthcare workers, where a reduction in depressive and anxiety symptoms was confirmed 14 months after the start of the pandemic (16).

This declining trend on mean values of psychopathological scales among healthcare workers after some time after the outbreak of pandemic may be related to improved control of the pandemic situation and increased knowledge of the course of the infection and its prevention. In addition, the reduction in anxiety may be associated with greater awareness of SARS-CoV-2, increased availability of personal protective equipment, and adherence to preventive measures, including disinfection and social distancing (17).

On the other hand, the evolving course of the pandemic, the emergence of new coronavirus variants, and the lack of effective treatment exacerbated the sense of frustration and helplessness (18). There are numerous indications of the longevity of the health effects caused by the pandemic (19). Moreover, fears of stigma and discrimination may hinder healthcare workers’ willingness to use psychotherapeutic interventions (18). According to research, many prefer to seek support from family and friends rather than professional psychological help (20).

Previous studies on the population of Polish healthcare workers have not taken into account the temporal evolution of the course of the pandemic. Therefore, the purpose of the present study was to assess the prevalence of depressive and anxiety symptoms and to subjectively evaluate the quality of life of healthcare workers during the different stages of the COVID-19 pandemic.

## Materials and methods

### Methodology

This is a Computer-Assisted Web Interview (CAWI) survey using a questionnaire distributed through social media (medical facebook groups). The survey was targeted at healthcare workers who lived and worked in Poland during the pandemic period. Participation in the survey was fully anonymous, and voluntary, and at each stage of the survey, respondents had the opportunity to opt-out of the study, without providing a reason. Before participating in the survey, respondents were informed about the nature of the study, its objectives and methodology, after which they gave their informed consent to participate. The survey was designed in four stages, which corresponded to the different waves of infections in Poland.

• Stage I, from April 17, 2020 to April 26, 2020–the daily number of cases ranged from 263 to 460 COVID-19 cases and 18–40 deaths;
• Stage II, from December 1, 2020 to December 30, 2020–the daily number of cases ranged from 2,921 to 14,835 cases and 29–620 deaths;
• Stage III, from March 20, 2021 to April 30, 2021–the daily number of cases ranged from 6,802 to 35,246 and from 428–954 deaths;
• Stage IV, from November 1, 2021 to November 31, 2021–the daily number of cases ranged from 9,839 to 29,062 and 209–793 deaths (21).

The survey was based on a questionnaire that consisted of several parts. The first included sociodemographic questions, including age, gender, place of residence, relationship status, medical profession and reduction in earnings. It also asked about past psychiatric history (before COVID-19 pandemic), including psychological and psychiatric consultation and drug treatment. Moreover, questions regarding to seeking additional information about COVID-19 and tracking statistics on COVID-19 were asked. The next section contained questions based on a 10-point Likert scale that asked about fear of contracting COVID-19, fear due to quarantine and neighbor isolation, and fear of infecting loved ones. The last part of the survey included three standardized psychometric tools to measure anxiety, depression and quality of life.

(1) Beck Depression Inventory II (BDI-II) a psychometric tool used to measure depression. It consists of 21 questions in which answers are classified from 0 to 3 points. Interpretation of the score depends on the number of points obtained. The following values were used as cutoff points: 0–11 points: no depression; 12–26: mild depression; 27–49: moderate depression; 50–63: severe depression (22–24). The polish version of scale was validated and revealed high reliability (25, 26).

(2) Generalized Anxiety Disorderd-7 (GAD-7)–is a seven-item tool for assessing generalized anxiety. Each question asks about the frequency of occurrence of certain psychological states in the past 14 days (0–not at all, 1–a few days, 2–more than half the time, 3–almost always). The analysis of the tool is based on the total score obtained, and the cutoff points were 5, 10, and 15 points, which correspond to mild, moderate and severe anxiety, respectively (27). Polish version of the scale was obtained from Patient Health Questionnaire Screeners (Pfizer–the owner of this questionnaires’ translations base) (28).

(3) Manchester Brief Assessment of Quality of Life (MANSA)–is a tool for assessing quality of life by evaluating 16 aspects of life. The 14 questions are based on a 7-point Likert scale (1–could not be worse, 7–could not be better). Four questions involve affirmative answers (two points) or denial (one point) of the occurrence of certain situations. The higher the total score, the higher the quality of life is rated, and the maximum possible number of points to be scored is 92 (29). The MANSA scale was constructed on the basis of the existing Lancashire Quality of Life tool Profiles (LQLP), which enables a comprehensive assessment of the quality of life (29). The MANSA scale is a condensed and slightly modified alternative that maintains psychometric parameters of the prototype (30). It was validated with satisfactory reliability in terms of internal consistency on Swedish population (31). The Polish version of the tool was prepared in the Department and Clinic of Psychiatry, Wrocław Medical University, in 2000.

The study was conducted in accordance with the Declaration of Helsinki and approval was obtained from the Bioethics Committee of the Medical University of Wrocław (approval number: KB-471/2020).

### Statistical analysis

The variables analyzed are qualitative, quantitative and ordinal. The Lilliefors test was used to assess normality of distribution, while the Brownian-Forsythe test was used to assess variance. Basic descriptive statistics were used to evaluate quantitative and ordinal variables. If the assumption of the equality of variance was not met, Welch’s ANOVA was performed. Subsequently, *post-hoc* tests were performed using the Games-Howell test. For qualitative variables, Pearson’s chi-square test with Bonferroni correction was used. Baseline linear models were used to assess the influence of sociodemographic variables on the results of the BDI-II, GAD-7, and MANSA scales.

A statistical significance level of <0.05 was assumed in each case. Statistical analysis was performed using Statistica 14.0 software from StatSoft.

## Results

### Materials

A detailed description of the study group is presented in Table 1. 1,243 healthcare workers participated in the survey during four waves of the pandemic in Poland. The largest number of respondents took part in the survey during wave 1 of the pandemic (632–50.9%). The vast majority were women (88.3%), people from large cities (47.8%) and those in a relationship (66.4%). The most common representatives of healthcare workers were medical doctors (37.6%). 13% of healthcare workers remarked that the pandemic had led to a reduction in their earning capacity, a percentage that decreased as the pandemic continued.

**TABLE 1:** Characteristics of the study group.

		Variable N (%)						
		Wave 1	Wave 2	Wave 3	Wave 4	Size effect	*p*	The whole group
Age (M ± SD)		36.48 ± 10.31	28.37 ± 8.85	31.47 ± 10.04	34.01 ± 10.64	0.108^a^	**0.001^c^**	33.84 ± 10.53
Sex	Male	60 (9.2)	39 (19.2)	31 (12.7)	15 (9.2)	0.114^b^	**0.001^d^**	145 (11.7)
	Female	572 (90.8)	164 (80.8)	214 (87.3)	148 (90.8)			1098 (88.3)
Place of residence	City of over 250,000 inhabitants	297 (47.0)	108 (53.2)	112 (45.7)	77 (47.3)	0.037^b^	0.818^d^	594 (47.8)
	City of 50,000–250,000 inhabitants	127 (20.1)	32 (15.8)	51 (20.8)	38 (23.3)			248 (20.0)
	Town of up to 50,000 inhabitants	98 (15.5)	31 (15.2)	38 (15.5)	23 (14.1)			190 (15.2)
	Rural area	110 (17.4)	32 (15.8)	44 (18.0)	25 (15.3)			211 (17.0)
Marital status	Married	360 (57.0)	35 (17.3)	83 (33.9)	80 (49.1)	0.186^b^	**<0.001^d^**	558 (44.9)
	In an informal relationship	111 (17.6)	65 (32.0)	63 (25.7)	28 (17.2)			267 (21.5)
	Single	161 (25.4)	103 (50.7)	99 (40.4)	55 (33.7)			418 (33.6)
Healthcare profession	Medical doctor	335 (53.0)	47 (23.2)	41 (16.7)	44 (27.0)	0.279^b^	**<0.001^d^**	467 (37.6)
	Nurse	173 (27.4)	34 (16.8)	93 (38.0)	51 (31.3)			351 (28.2)
	Other	124 (19.6)	122 (60.0)	111 (45.3)	68 (41.7)			425 (34.2)
Prior psychiatric treatment (before COVID-19 pandemic)	Yes	115 (18.2)	37 (18.2)	42 (17.1)	22 (13.5)	0.041^b^	0.548^d^	216 (17.4)
	No	517 (81.8)	166 (81.8)	203 (82.9)	141 (86.5)			1027 (82.6)
Psychiatric drug treatment	Yes	103 (16.3)	31 (15.3)	39 (15.9)	20 (12.3)	0.036^b^	0.649^d^	193 (15.5)
	No	529 (83.7)	172 (84.7)	206 (84.1)	143 (87.7)			1050 (84.5)
Limitation of earning capacity	Yes	101 (16.0)	26 (12.8)	24 (9.8)	11 (6.8)	0.102^b^	0.061^d^	162 (13.0)
	No	531 (84.0)	177 (87.2)	221 (90.2)	152 (93.2)			1081 (87.0)
Seeking information about COVID-19	Yes	470 (74.4)	113 (55.7)	114 (46.5)	104 (63.8)	0.234^b^	**<0.001^d^**	801 (64.4)
	No	162 (25.6)	90 (44.3)	131 (53.5)	59 (36.2)			442 (35.6)
Tracking statistics on COVID-19	Yes	407 (64.4)	117 (57.6)	120 (49.0)	87 (53.4)	0.127^b^	**<0.001^d^**	731 (58.8)
	No	225 (35.6)	86 (42.4)	125 (51.0)	76 (46.6)			512 (41.2)
Pandemic wave	1	–	–	–	–		–	632 (50.9)
	2	–	–	–	–		–	203 (16.3)
	3	–	–	–	–		–	245 (19.7)
	4	–	–	–	–		–	163 (13.1)

^a^*ε*^2^.

^b^Cramer’s V.

^c^Kruskal–Wallis test.

^d^Chi-squared test. Significant differences (*p* < 0.05) were marked with bold characters.

### Interpretation of the Beck Depression Inventory II, Generalized Anxiety Disorderd-7, and Manchester Brief Assessment of Quality of Life scales over four waves among healthcare workers

**FIGURE 1:**
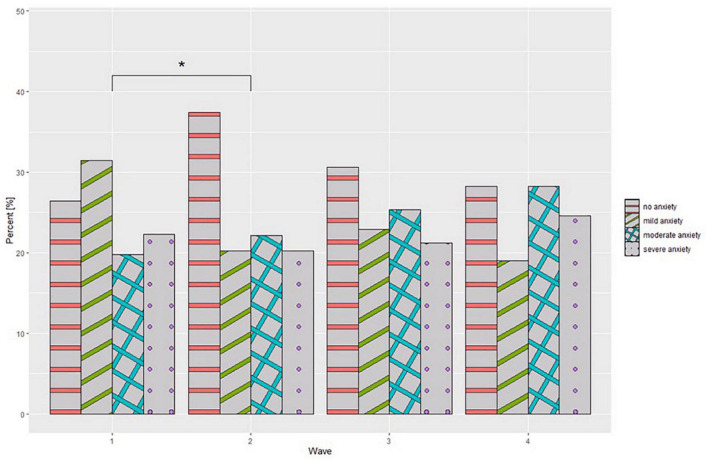
Generalized Anxiety Disorderd-7 (GAD-7) interpretation at different stages of the study. *p < 0.05.

**FIGURE 2:**
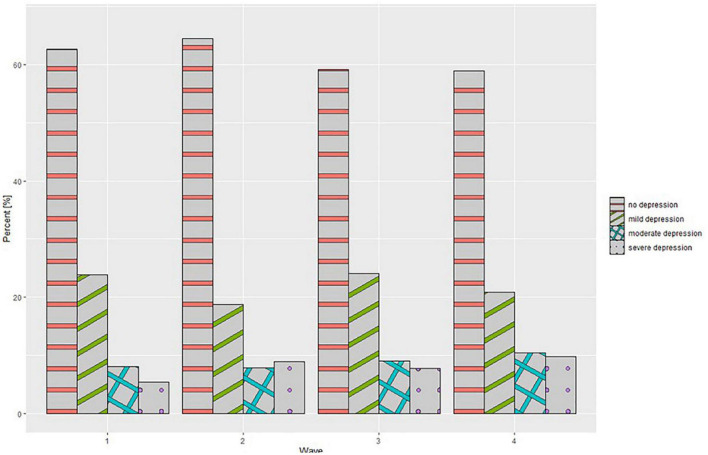
Beck Depression Inventory II (BDI-II) interpretation at different stages of the study.

A detailed comparison of the BDI-II, GAD-7, and MANSA scales is presented in Table 2. The ANOVA type II test of mean values between waves showed no significant statistical differences for each of the scales- the BDI-II (*p* = 0.316), GAD-7 (*p* = 0.245), and MANSA (*p* = 0.413). Analysis of the GAD-7 scale interpretation showed statistically significant differences (*p* = 0.001). As the pandemic continued, a gradual increase was observed in the percentage of healthcare workers whose scale scores indicated the presence of moderate anxiety and severe anxiety. In a *post hoc* analysis (Games-Howell test), significant changes were observed only between wave 1 and wave 2 of the pandemic (*p* = 0.017) as it is shown on Figure 1. Analysis of the BDI-II scale interpretation showed no statistically significant differences (*p* = 0.001) (Figure 2). As the COVID-19 pandemic progressed, no significant changes were also observed in the healthcare workers’ quality of life scores. Moreover, a detailed comparison of the BDI-II, GAD-7, and MANSA scales taking into account medical professions is presented in Table 3. No significant differences were found other than differences the mean BDI-II scale scores between waves for “other medical professions.”

**TABLE 2:** Comparison of the BDI-II, GAD-7, and MANSA scales in relation to the different stages of the study.

Variable		Wave 1 N (%)	Wave 2 N (%)	Wave 3 N (%)	Wave 4 N (%)	Power of a test	Size effects	*p*
BDI-II M ± SD		10.27 ± 8.48	10.52 ± 9.34	11.09 ± 8.54	11.72 ± 10.78	0.959	0.003^a^	0.316^c^
BDI-II interpretation	No depression	396 (62.7)	131 (64.5)	145 (59.2)	96 (58.9)		0.051^b^	0.392^d^
	Mild depression	151 (23.9)	38 (18.7)	59 (8.9)	34 (20.9)			
	Moderate depression	51 (8.0)	16 (7.9)	22 (9.1)	17 (10.4)			
	Severe depression	34 (5.4)	18 (8.9)	19 (7.8)	16 (9.8)			
GAD-7 M ± SD		9.10 ± 5.99	8.45 ± 6.06	8.86 ± 6.01	9.77 ± 6.55	0.998	0.004^a^	0.245^c^
GAD-7 interpretation	No anxiety	167 (26.4)	76 (37.4)	75 (30.6)	46 (28.2)		0.084^b^	**0.001** ^d^
	Mild anxiety	199 (31.5)	41 (20.2)	56 (22.9)	31 (19.0)			
	Moderate anxiety	125 (19.8)	45 (22.2)	62 (25.3)	46 (28.2)			
	Severe anxiety	141 (22.3)	41 (20.2)	52 (21.2)	40 (24.6)			
MANSA M ± SD		62.10 ± 11.98	63.54 ± 12.12	63.11 ± 11.62	63.03 ± 14.47	0.704	0.002^a^	0.413^c^

^a^*ε*^2^.

^b^Cramer’s V.

^c^ANOVA type II.

^d^Chi-squared test. M, mean; SD, standard deviation. Significant differences (*p* < 0.05) were marked with bold characters.

**TABLE 3:** Comparison of the BDI-II, GAD-7, and MANSA scales in relation to the different stages of the study for each medical profession.

Variable		Wave 1 N (%)	Wave 2 N (%)	Wave 3 N (%)	Wave 4 N (%)	Power of a test	Size effects	*p*
**Medical doctors (*N* = 457)**								
BDI-II M ± SD		10.79 ± 8.7	9.57 ± 7.8	11.6 ± 9.4	11.11 ± 9.8	0.871	0.007^a^	0.347^c^
BDI-II interpretation	No depression	74 (59.7)	85 (69.7)	65 (58.6)	40 (58.8)		0.439^b^	0.276^d^
	Mild depression	35 (28.2)	20 (16.4)	22 (19.8)	15 (22.1)			
	Moderate depression	8 (6.4)	12 (9.8)	14 (12.6)	7 (10.3)			
	Severe depression	7 (5.7)	5 (4.1)	10 (9.0)	6 (8.8)			
GAD-7 M ± SD		8.91 ± 6.26	8.23 ± 5.95	8.57 ± 6.01	9.22 ± 6.69	0.982	0.003^a^	0.715^c^
GAD-7 interpretation	No anxiety	38 (30.7)	47 (38.5)	37 (33.3)	21 (30.9)		0.054^b^	0.763^d^
	Mild anxiety	34 (27.3)	26 (21.3)	24 (21.6)	12 (17.7)			
	Moderate anxiety	25 (20.2)	26 (21.3)	28 (25.2)	20 (29.3)			
	Severe anxiety	27 (21.8)	23 (18.9)	22 (19.9)	15 (22.1)			
MANSA M ± SD		61.54 ± 12.33	64.20 ± 11.11	62.0 ± 11.72	64.28 ± 14.74	0.969	0.001^a^	0.226
**Nurses (*n* = 351)**								
BDI-II M ± SD		10.55 ± 8.95	11.71 ± 10.58	10.12 ± 7.79	11.84 ± 10.35	0.711	0.005^a^	0.163^c^
BDI-II interpretation	No depression	109 (63.0)	18 (52.9)	56 (60.2)	31 (60.8)		0.085^b^	0.567^d^
	Mild depression	37 (21.4)	10 (29.4)	27 (29.0)	10 (19.6)			
	Moderate depression	14 (8.1)	1 (2.9)	5 (5.4)	5 (9.8)			
	Severe depression	13 (7.5)	5 (14.8)	5 (5.4)	5 (9.8)			
GAD-7 M ± SD		9.55 ± 6.18	8.94 ± 6.15	8.94 ± 6.09	10.97 ± 5.88	0.998	0.012^a^	0.271^c^
GAD-7 interpretation	No anxiety	46 (26.7)	13 (38.2)	28 (30.1)	8 (15.7)		0.098^b^	0.267^d^
	Mild anxiety	49 (28.3)	4 (11.8)	22 (23.7)	14 (27.5)			
	Moderate anxiety	35 (20.1)	9 (26.5)	24 (25.8)	15 (29.3)			
	Severe anxiety	43 (24.9)	8 (23.5)	19 (20.4)	14 (27.5)			
MANSA M ± SD		61.22 ± 12.52	62.35 ± 12.82	63.62 ± 12.06	60.57 ± 13.69	0.841	0.008^a^	0.417^c^
**Others medical professions (*n* = 467)**								
BDI-II M ± SD		9.92 ± 8.15	12.14 ± 11.61	11.93 ± 7.79	12.52 ± 12.77	0.948	0.013^a^	**0.011^c^**
BDI-II interpretation	No depression	213 (63.6)	28 (59.8)	24 (58.5)	25 (58.6)		0.103^b^	0.174^d^
	Mild depression	79 (23.6)	8 (17.0)	10 (24.4)	9 (20.6)			
	Moderate depression	29 (8.7)	3 (6.4)	3 (7.3)	5 (11.4)			
	Severe depression	14 (4.1)	8 (17.0)	4 (9.8)	5 (11.4)			
GAD-7 M ± SD		8.93 ± 5.77	8.68 ± 6.38	9.41 ± 5.96	9.25 ± 6.98	0.358	0.001^a^	0.930^c^
GAD-7 interpretation	No anxiety	83 (24.8)	16 (34.0)	10 (24.4)	17 (38.6)		0.099^b^	0.085^d^
	Mild anxiety	116 (34.6)	11 (23.4)	10 (24.4)	5 (11.4)			
	Moderate anxiety	65 (19.4)	10 (21.3)	10 (24.4)	11 (25.0)			
	Severe anxiety	71 (21.2)	10 (21.3)	11 (26.8)	11 (25.0)			
MANSA M ± SD		62.76 ± 11.56	62.68 ± 14.48	64.91 ± 10.20	63.95 ± 14.88	0.798	0.003^a^	0.697^c^

^a^*ε*^2^.

^b^Cramer’s V.

^c^ANOVA type II.

^d^Chi-squared test. M, mean; SD, standard deviation. Significant differences (*p* < 0.05) were marked with bold characters.

### Anxiety due to quarantine, isolation of a neighbor, and from one’s own illness

**FIGURE 3:**
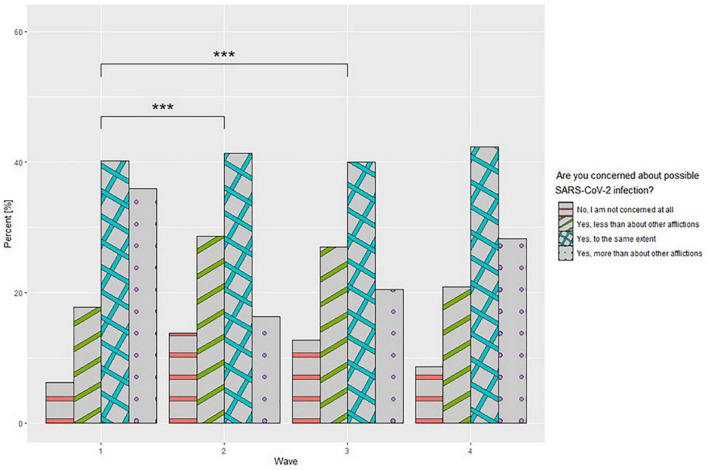
Fear of COVID-19 infection concerning other conditions at different stages of the study. ***p < 0.001.

Questions based on a 10-point Likert scale were used to assess the fear of one’s own, as well as a neighbor’s disease and quarantine. Detailed data for this part of questionnaire are presented in Table 4. Significant differences were observed in ANOVA type II test between waves in each question, with the highest values achieved in wave 1 of the pandemic. Games-Howel *post-hoc* tests showed that there was a significant reduction (*p* < 0.001) in concern between waves 1 and 2 and 1 and 3 as the pandemic continued for each question. Furthermore, a significant increase (*p* < 0.001) was observed between wave 3 (mean value–3.09) and wave 4 (3.26) for fear of one’s disease and that of a neighbor’s disease. An analogous relationship (*p* = 0.048) was observed for adherence to government recommendations to combat the pandemic with following mean values for waves 3 (7.87) and 4 (8.25). In addition, the Pearson correlation coefficient revealed a relationship between adherence to government recommendations and fear of getting sick (*r* = 0.34; *p* < 0.001), fear of a neighbor’s disease (*r* = 0.263; *p* < 0.001) and its quarantine (*r* = 0.23; *p* < 0.001). For the question assessing the level of concern for COVID-19 concerning individual diseases, it was shown that between waves 1 and 2 and waves 1 and 3 of the pandemic, there was an increase in those who were not concerned about COVID-19 and a significant decrease in those who were more concerned than other diseases (Figure 3).

**TABLE 4:** Comparison of the mean values of the assessment of fear of disease, fear due to neighbor’s disease and neighbor’s quarantine, and adherence to government recommendations for each wave of the pandemic.

	Wave 1	Wave 2	Wave 3	Wave 4	*p*
**Anxiety about being infected with COVID-19 disease**					
Mean	6.07	5.3	5.2	5.9	**<0.001^a^**
Comparison of individual COVID-19 pandemic waves			x	x	**<0.001^b^**
		x		x	**<0.001^b^**
		x	x		0.997^b^
	x			x	0.999^b^
	x		x		0.066^b^
	x	x			**0.019^b^**
**Anxiety about neighbors being infected with SARS-CoV-2**					
Mean	4.89	3.09	3.09	3.26	**<0.001^a^**
Comparison of individual COVID-19 pandemic waves			x	x	**<0.001^b^**
		x		x	**<0.001^b^**
		x	x		**<0.001^b^**
	x			x	0.934^b^
	x		x		0.389^b^
	x	x			**0.048^b^**
**Anxiety about neighbors in quarantine**					
Mean	3.84	2.57	2.62	2.62	**<0.001^a^**
Comparison of individual COVID-19 pandemic waves			x	x	**<0.001^b^**
		x		x	**<0.001^b^**
		x	x		**<0.001^b^**
	x			x	0.999^b^
	x		x		0.999^b^
	x	x			0.999^b^
**Adherence to the Ministry of Health recommendations regarding SARS-CoV-2 prevention**					
Mean	8.92	8.13	7.87	8.25	**<0.001^a^**
Comparison of individual COVID-19 pandemic waves			x	x	**<0.001^b^**
		x		x	**<0.001^b^**
		x	x		**<0.001^b^**
	x			x	0.934^b^
	x		x		0.389^b^
	x	x			**0.048^b^**

^a^ANOVA type II.

^b^Games-Howell *post-hoc* test. Significant differences (*p* < 0.05) were marked with bold characters.

### Relationships between sociodemographic variables among healthcare workers and Beck Depression Inventory II, Generalized Anxiety Disorderd-7, and Manchester Brief Assessment of Quality of Life scales

A detailed summary of the relationships between sociodemographic variables and the mean values of the BDI-II, GAD-7, and MANSA scales based on linear models is presented in Table 5. In the analysis of healthcare workers, it was shown that the mean value of the BDI-II and GAD-7 scales statistically significantly (*p* < 0.001) decreases with increasing age. In addition, women score higher on both scales. It was also shown that healthcare workers who are not in a relationship score higher on the BDI-II scale. Importantly, both the limitation of earning capacity, previous psychiatric treatment, and tracking COVID-19 statistics and seeking information significantly increases the mean scores of the BDI-II and GAD-7 scales.

**TABLE 5:** Summary of relationships between sociodemographic variables and the mean values of the BDI-II, GAD-7, and MANSA scales based on linear models.

		BDI-II				GAD-7				MANSA			
		Value	SD	*t*	*p*	Value	SD	*t*	*p*	Value	SD	*t*	*p*
Age		–0.100	0.025	–3.99	** < 0.001**	–0.061	0.017	–3.60	** < 0.001**	0.036	0.034	1.05	0.293
Sex	Male	–1.690	0.802	–2.11	**0.035**	–2.506	0.544	–4.65	** < 0.001**	1.455	1.101	1.32	0.187
Place of residence	Rural	–1.131	0.717	–1.58	0.115	–0.338	0.487	–0.693	0.488	–0.196	1.373	–0.14	0.886
	Town of up to 50,000 inhabitants	–2.102	0.746	–2.82	**0.004**	–0.350	0.507	–0.691	0.489	0.548	1.438	0.38	0.702
	City of over 250,000 inhabitants	–0.599	0.677	–0.88	0.376	–0.026	0.461	–0.056	0.955	–1.937	1.30	–1.48	0.137
Marital status	Single	0.885	0.365	2.42	**0.016**	0.287	0.250	1.15	0.249	–1.464	0.501	–2.92	**0.003**
	In an informal relationship	0.512	0.411	1.24	0.213	0.156	0.280	0.56	0.576	0.09	0.565	0.17	0.861
Healthcare profession	Nurse	0.065	0.377	0.17	0.921	0.471	0.255	1.84	0.065	–0.723	0.517	–1.39	0.162
	Other	0.036	0.359	0.09	0.921	–0.384	0.243	–1.58	0.113	0.265	0.492	0.53	0.584
Limitation of earning capacity	Yes	3.391	0.754	4.50	** < 0.001**	1.601	0.513	3.12	**0.001**	–5.337	1.03	–5.18	< 0.001
Prior psychiatric treatment (before COVID-19 pandemic)	Yes	2.854	0.667	4.28	** < 0.001**	1.888	0.452	4.17	** < 0.001**	–3.401	0.917	–3.71	** < 0.001**
Seeking information about COVID-19	Yes	0.795	0.265	3.01	**0.003**	0.826	0.178	4.63	** < 0.001**	–0.068	0.364	–0.19	0.852
Tracking statistics on COVID-19	Yes	0.873	0.257	3.39	** < 0.001**	0.934	0.73	5.39	** < 0.001**	0.134	0.355	0.38	0.704

Significant differences (*p* < 0.05) were marked with bold characters.

### Internal validity of the scales

Each scale used in the study revealed high internal validity. The following Cronbach’s alpha values were obtained: 0.912 for BDI-II, 0.929 for GAD-7, and 0.852 for MANSA.

## Discussion

The study found significant differences between the waves of the pandemic in terms of the mental condition of healthcare workers as the pandemic continued. Changes included an increase in the percentage of people suffering from anxiety disorders. Compared to a similar study among the general Polish population, there were significantly lower levels of depression (no depression: 63.7% vs. 50.1%) for the first wave of the pandemic, but slightly higher anxiety (no anxiety in: 26.4% vs. 28.9%). Additionally, healthcare workers rated their quality of life better than the rest of the population (mean value 62.1 vs. 60.65 on the MANSA scale–wave I) (32).

High exposure and direct contact with the pathogen may have influenced the heightened anxiety in healthcare workers. This situation increased the risk of infection, which is estimated to be up to three times higher than in the general population (33). In particular, at the beginning of the pandemic, when the level of knowledge about the disease was low, there was no protective vaccination and no effective treatment available (34, 35). Although the proportion of moderate and severe anxiety increased with successive waves, in the first wave it was the smallest proportion of respondents who reported a lack of clinical anxiety. Significant shortages of personal protective equipment supplies were reported at the beginning of the pandemic. In addition, due to deficits in medical equipment such as ventilators, healthcare workers were unable to provide adequate care to all patients, which resulted in frustration and anxiety (36, 37). At the same time healthcare workers were afraid of infecting their loved ones, there were also problems with a place to quarantine in case of infection (10, 38). Increasing anxiety in successive waves may also have been related to delayed psychiatric reactions to overwhelming clinical workloads (11). However, the increase in anxiety was not related to fear of getting sick themselves or those around them. On the contrary, these fears were rated lower in subsequent waves in our observations. A similar phenomenon occurred among staff working in an emergency department (ED) in Singapore (17). The decline may have been due to the increase in the availability of personal protective equipment, immunizations and effective treatment for the cause of the disease. In this point, it would be worth comparing the results to another study among Polish healthcare workers that also used the GAD-7 scale and the same cut-offs. It showed a lower recognition of anxiety (45% vs. 62.6 to 73.6% depending on wave in this study), but the mean age of the respondents was much higher (mean 44.44 vs. 33.84 in this study), which is recognized as a protective factor in epidemiological studies (39).

Analogous to the results of this study changes in psychopathology, were shown in Argentina. In a longitudinal study among healthcare workers there, the prevalence of depressive or anxiety disorders increased (from 46 to 63%) on the Kessler Psychological Distress Scale a few months after the pandemic outbreak (40). In contrast, a study among ED workers in Singapore showed different trends. After 1 year of the pandemic, there was a decrease in anxiety and an increase in depressive symptoms. However, it should be mentioned, that the percentage of clinically significant depression among healthcare workers in Singapore was much lower at the beginning of the pandemic than in our study (25.3% vs. 37.3%). Their increase in depressive symptoms could be contributed to staff shortages and extended work hours which additionally proved to be more exhausting than before. Concurrently, a reduction in anxiety was associated with the development of guidelines for managing patients, as well as the implementation of immunizations (17). In an Australian cross-sectional study, healthcare workers in the second wave of the pandemic scored higher than in the first wave on the Depression, Anxiety and Stress Scale (DASS-21). They revealed an increase in the level of workplace conflicts, as well as difficulties in taking leave, among the significant reasons for the deterioration (41). Interesting results were presented by a study that showed among acute care healthcare workers the impact of work-related sense of coherence (W-SoC) on psychopathology. The study concluded that during the first 3 months high W-SoC was associated with milder symptoms of depression and trauma, but after 1 year of the study, W-SoC among these respondents declined and ceased to be a protective factor (42).

It is worth noting that the average score on the quality of life scale did not change among healthcare workers in each wave. One factor contributing to this may be the relatively high financial bonuses for healthcare workers in Poland. In comparison, the subjective assessment of the quality of life of the Polish population with successive waves received lower scores which were related to their reduced financial satisfaction (32). It is vital to mention that quality of life as assessed by the MANSA scale takes into account many aspects such as physical and mental health, financial and sexual satisfaction, or the quality of social and family relationships, hence a potential increase in financial satisfaction may offset deficits in other issues (29). Another study using the Professional Quality of Life-5 scale found an imbalance between job satisfaction (compassion satisfaction) and work overload (compassion fatigue) as a contributing factor to reduced quality of life (43).

While analyzing the results, it should be considered that the Polish healthcare system is facing underfunding and staff shortages. According to Eurostat, in Poland, 4.8% of GDP was spent from public money to the healthcare system, which is one of the smallest amounts in the European Union (EU) (44). The number of medical doctors and nurses per 100,000 of months is also critical, at 2.4 and 5.1, respectively (45, 46). Significantly due to the nature of the survey, Poland is penultimate in the European Union in terms of the number of psychiatrists (9 per 10,000 population) (47). In the Organization for Economic Cooperation and Development’s report “Health at glance: Europe 2020” Poland also ranked penultimate in the EU in terms of public satisfaction with the quality of health services provided (48). This had a demotivating effect on Polish healthcare workers during the pandemic in the form of conspiracy theories and lack of adherence to medical recommendations, which devalued their work (49).

The influence of socio-demographic variables on scale scores in this study reflects trends in other populations. Specifically, among studies of healthcare workers from other countries, women and younger people also showed more severe depressive and anxiety symptoms as measured by the same scale (GAD-7) (40, 50). It is consistent with the concept that people with more life experience show better mental resilience and emotional regulation (51). In contrast, the lower resilience of women than men to stress and the resulting psychiatric complications during the pandemic have been linked to environmental, psychodynamic, cognitive and physiological moderators (e.g., ovarian hormone fluctuations) (52). It is not surprising that healthcare workers with a prior history of psychiatric disorders have more severe psychopathological symptoms (40). However, contrary to intuition, being in a partnership relative to being single in many studies has not been a significant moderator of psychopathology scale scores, and in our study it was significant in the context of depression and quality of life, but not anxiety intensity (15, 53). An unfavorable relationship in the context of mental health was also found between the increased frequency of searching for information about the pandemic on the Internet and tracking statistics on the Internet, as confirmed by the results of another Polish study (53). Analyses of the quality of media coverage showed that audiences were particularly vulnerable to disinformation and conspiracy theories during the pandemic (54). More interestingly, searches for depression and suicide, but not for anxiety disorders, declined during the pandemic’s peak in illnesses and deaths on search engines (55). Other studies have cited having children and maintaining good relationships with friends as protective factors against mental disorders during a pandemic (17). Attention should be paid to the fact that in the linear models no significant differences were found between the professions and the results of the scales used. At the same time, there is a large disproportion of the respondents’ medical professions distribution between the study stages. To check whether the dominance of any of them biased the overall trends shown in the study, a wave-to-wave analysis was additionally performed for each profession that excluded such limitation. In studies from other countries according to depression, anxiety and insomnia scales, nursing profession appeared to be the most burdened among other medical professions (50, 56).

The survey has several limitations. First, due to the online method of distributing the surveys, the number of people reached is unknown. Second, we do not have access to the percentage of respondents withdrew from the survey during completion. The results of our scales may be underestimated because people with severe mental disorders are less likely to participate in surveys (57). There was also be a significant disproportion in the number of survey respondents between survey stages with a decreasing trend. Due to cross-sectional methodology of the study on disparate groups of respondents, no direct conclusions can be drawn about the evolution of recorded changes in symptom intensity. Moreover, the survey sample is not representative in terms of gender and age, nor of the or the structure of employment in the polish healthcare system. The vast majority of women may lead to overdiagnosis in the epidemiological assessment of mental disorders in this population (58). Also, the various medical professions among the respondents were not distinguished, nor whether healthcare workers were required to work with patients with COVID-19, which significantly affects the results of the research (59). Due to the anonymous nature of the questionnaire and the way it was distributed, it was impossible to provide psychological care to those exposed, but the mere fact of participation could force self-reflection on one’s own mental condition, which is a positive predictor of taking effective treatment (60). Another limitation is the lack of validation in the literature of the Polish translation of the GAD-7 and MANSA scales, which may undermine the reliability of the results.

Given the particular exposure to mental stress, healthcare workers should be provided with extensive access to psychological and psychiatric care. Public hospitals should provide such care as a compensation to their employees. It is also worth considering dedicated training in stress management for medical staff (61). Other countries have also proven successful methods, such as team support sessions, peer support programs, mental health and wellness programs, a palliative support team, philosophical services and clergy support. In summary, the most common coping styles were emotional support, planning, and active coping (15, 62). Given the high burden of stress, it would also be worthwhile to provide early intervention among healthcare workers for the prevention of post-traumatic stress disorder (63).

## Conclusion

Based on the experience developed in previous pandemic waves, the healthcare system’s crisis management model should be improved for new epidemiological threats in the future (64, 65). This is particularly important given that lack of mental health hygiene among healthcare workers promotes professional burnout and adversely affects the quality of healthcare delivery (66).

The COVID-19 pandemic is significantly affecting the mental health and quality of life of healthcare workers, a trend that is not uniform. Significant increases in anxiety symptoms, especially moderate and severe anxiety, were observed between the first waves of the study. Women, single people and those with a psychiatric history are more likely to be affected by the destructive impact of the pandemic. Given the ongoing situation, it is necessary to provide longitudinal studies on the mental health of medical workers, who are the most important link in the fight against the pandemic.

## Data availability statement

The raw data supporting the conclusions of this article will be made available by the authors, without undue reservation.

## Ethics statement

The studies involving human participants were reviewed and approved by the Bioethics Committee of the Medical University of Wrocław. The patients/participants provided their written informed consent to participate in this study.

## Author contributions

MB, KK, BB, and AM-M: conceptualization, methodology, writing—original draft, and writing—review and editing. KK: formal analysis. MB: funding acquisition and visualization. MB, BB, and AM-M: investigation and supervision. All authors contributed to the article and approved the submitted version.
